# Advanced Mid-Infrared Sensors for Molecular Analysis

**DOI:** 10.1021/acs.analchem.4c06799

**Published:** 2025-03-28

**Authors:** João Flávio da Silveira Petruci, Danielle da Silva Sousa, Boris Mizaikoff

**Affiliations:** † Institute of Chemistry, Federal University of Uberlândia (UFU), Uberlândia, MG 38400-902, Brazil; ‡ Institute of Analytical and Bioanalytical Chemistry, Ulm University, 89081 Ulm, Germany; § Hahn-Schickard, 89077 Ulm, Germany

## WHY SENSORS IN THE MID-INFRARED RANGE?

Among the diverse family of (bio)chemical optical sensors based on light absorption, the mid-infrared (MIR) spectral range (i.e., covering wavelengths from 2 to 25 μm) has emerged as an exceptional choice, due to the inherent highly discriminatory qualitative and quantitative molecular information.^1^ The excitation of fundamental vibrational, rotational, and vibro-rotational transitions excited within organic and inorganic molecules across the liquid, solid, and gas phase generates a distinctive spectrum (also known as fingerprint) for each sample. These unique MIR spectral signatures lend themselves to the development of highly selective chem/bio sensor systems with superior selectivity, especially when compared to absorption-based sensors operating in the ultraviolet-to-visible (UV/vis) and near-infrared (near-IR) spectral regime. MIR spectroscopy is a nondestructive, label-free analytical technique widely adopted across academic/research and industrial/practical application scenarios. The versatility of MIR spectroscopy has been demonstrated in areas such as medical diagnostics,^2,3^ environmental monitoring,^4^ energy research,^5^ and the food and agriculture industries.^6^ Consequently, the translation of conventional—usually laboratory-based—IR spectroscopy into compact, portable, and robust chem/biosensing and diagnostic tools motivates this comprehensive review on emerging MIR technologies advancing key sensor components and augmented promising analytical application and deployment scenarios.

Optical sensing systems usually rely on three key components: (a) a light source, (b) a sample compartment/interface made from materials that transmit the incident radiation enabling interaction between photons and samples (also known as a active optical transducer), and (c) a detector.^7^ Related spectroscopic techniques such as UV/vis, and near-IR spectroscopy benefited from significant technological advancements between the 1960s and 1980s, leading to their rapid evolution toward miniaturized and cost-effective sensing systems for analytical applications in the liquid, solid, and gas phase. While these techniques have reached a level of maturity that facilitates miniaturized and on-chip analytical devices, direct sensing using UV/vis/NIR spectroscopy is fundamentally limited by spectral overlap in complex real-world samples frequently resulting in ambiguous analytical signals. In contrast, the MIR spectral region offers inherent selectivity without requiring additional chemical interactions (i.e., such as indirect methods using chemical dyes, etc.) for providing discriminatory analytical signatures. Until the early 21st century, the classical application of MIR-based spectroscopy was largely focused on molecular structure investigations rather than real-world qualitative or quantitative analyses.^8^ This may be attributed to several challenges associated with MIR components including (i) the miniaturization of light sources (e.g., laser diodes) capable of providing sufficient optical output power, long lifetimes, and operational stability; (ii) the limited transparency of common optical materials across the MIR spectrum facilitating efficient radiation propagation through cuvettes, optical fibers, or other waveguiding structures; (iii) the strong interference water, the most common background molecule/interferant in gas and liquid phase real-world samples; and (iv) the potential for overall device and component miniaturization and integration.

Significant advancements in microfabricated and nanofabricated optical components have recently paved the way for “on-chip” integrated MIR sensing platforms.^9^ These miniaturized systems have demonstrated that parameters such as sensitivity, robustness, portability, and user-friendliness are not limited to laboratory-scale instrumentation.^10,11^ While detailed reviews on individual components used in MIR-based sensors document the progress in each technical area,^1,12,13^ the present review authoritatively aims at providing a comprehensive overview on the state-of-the-art and advancements for components specifically relevant to the evolution of MIR sensing systems during the past five years. Derived from over 100 research articles, key developments toward “on-chip” MIR sensors for analytical chemistry will be highlighted. Following a brief overview of the most commonly used sensing modalities, recent innovations in MIR technology focusing on light sources, waveguides, and detectors will be summarized. Finally, selected contemporary application examples for analyzing liquid- and gas-phase samples ranging from medical diagnostics to food analysis will augment the utility of MIR sensing schemes.

## OPTICAL SENSING MODALITIES

### Attenuated Total Reflection (ATR): General Concepts

Infrared spectroscopy based on attenuated total reflection (ATR)—also known as evanescent field absorption spectroscopy—relies on the principle of total internal reflection (TIR), and has been widely used for analyzing liquid, thin-film, semisolid, and solid samples.^14^ When radiation is introduced at a specific angle into an internal reflection element (IRE)—made from a material with a high refractive index—from an adjacent medium (i.e., surrounding environment) with a lower refractive index, light propagates within the waveguide via total internal reflection. However, a portion of the radiation penetrates into the surrounding low-refractive-index medium, establishing an evanescent field with an intensity that exponentially decreases with increasing distance from the waveguide surface. In the MIR range, the evanescent field is wavelength-dependent and typically penetrates only a few hundred nanometers to a few micrometers into the optically rarer medium (e.g., sample) rendering ATR spectroscopy a quasi surface-sensitive technique.^13^ The penetration depth (*d*_p_) of the evanescent field depends on the refractive index of the waveguide (*n*_wg_), the refractive index of the adjacent medium (*n*_c_), the angle of incidence (θ), and the wavelength of the incident light (λ), and may be calculated as described using eq 1:^15^
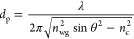
1

If a sample is added at the IRE surface (i.e., waveguide), analyte molecules within the penetration depth interact with the evanescent field generated at the surface with intensity *I*_0_. This interaction leads to light absorption resulting in a reduced light intensity (*I*), and consequently, light attenuation corresponding to specific vibrational, rotational, and vibro-rotational transitions of the molecules. As a result, an IR-ATR spectrum is recorded, and the absorption (*A*) of the evanescent field follows a modified Beer–Lambert relationship,^9^ as shown in eq 2, where *r* is the fraction of radiative energy residing outside the waveguide (i.e., within the evanescent field), ε the molar absorptivity, and *c* the concentration. This relationship allows for the quantification of analytes under investigation, similar to a transmission–absorption experiment.
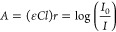
2

Consequently, in any absorption-based analytical measurement, the signal-to-noise ratio (SNR) is the critical parameter for enhancing the analytical sensitivity, as it directly influences the limits of detection (LoD) and quantification (LoQ). Conversely, the intensity of the emitted light from the radiation source only plays an indirect role, i.e., the magnitude of the useful analytical signal relies on the number of photons that have interacted with molecules delivering analytical information to the detector versus photons reaching the detector without sample interaction giving rise to background noise. Hence, intimate, reliable, and reproducible interaction of photons with sample constituents at the transducer is crucial to maximizing the SNR. In ATR-based spectroscopy taking advantage of evanescent field absorption, the SNR strongly depends on the dimensions, geometry, material (i.e., optical properties), and structure of the waveguide. If the waveguide thickness is significantly larger than the wavelength of the propagating radiation, total internal reflection (TIR) occurs only a few times along the long axis of the waveguide, thereby limiting the interaction at the waveguide–sample interface to a few hotspots. Consequently, reducing the waveguide thickness significantly improves the analytical sensitivity. The structure and composition of the waveguide material also play key roles in determining the analytical signal. While commonly used optical materials in UV/vis, and near-IR applications lack sufficient transparency across the majority of the MIR spectrum, recent advances have identified promising alternatives. For example, gallium arsenide on aluminum gallium arsenide (GaAs/AlGaAs),^16^ germanium-on-silicon (Ge-on-Si),^17^ or nanocrystalline diamond on silicon (NCD-onSi)^18^ platforms have been considered to be among the most promising material systems in waveguide-based MIR sensor technology. Waveguides fabricated from chalcogenide glasses and purely silicon-based materials have also been designed for on-chip sensing applications. Beyond the waveguide, a MIR light source operating in continuous or pulsed mode, optical components such as lenses, mirrors, and/or coupling fiberoptics, and a detector constitute the essential experimental setup for IR-ATR spectroscopy and sensing. These components are discussed in detail within the corresponding sections.

### Transmittance/Absorbance Spectroscopy: General Concepts

Conventional molecular absorption spectroscopy correlates the light absorbed by molecules at a specific wavelength with their concentration in a specific sample.^19^ The Beer–Lambert law describes the relationship between the intensity of transmitted light (*I*) after passing through the sample and the intensity of the incident light (*I*_0_).^20^ This relationship is determined by the analyte concentration (*C*), the absorption coefficient of the sample at a specific wavelength (α(λ)), and the optical path length (*L*), as expressed in eq 3:
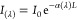
3

If gaseous samples are considered, the absorption cross-section (σ) is a wavelength-dependent parameter used to quantify a species ability to absorb light.^7^ σ relates to the concentration and the absorption coefficient as follows:
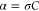
4

Since the molecular absorption depends on temperature and pressure, the resulting line shape has to be considered.^21^ To accurately determine the absorbance, the absorption profile is derived as an integral, as shown in eq 5. Consequently, the analyte concentration is ultimately related to the absorption coefficient and the optical path length, as described in eq 6.
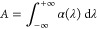
5
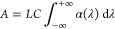
6

The instrumentation for conventional benchtop absorption spectroscopy—now usually Fourier-transform infrared (FTIR) systems—typically includes a light source (i.e., a broadband radiation emitter for multianalyte determination), a wavelength selector (e.g., interferometer, prism, diffraction grating, etc.), a sample compartment with a defined optical path length (e.g., cuvette for liquids, multipass cell for gases, etc.), and a detector (e.g., deuterated triglycine sulfate (DTGS), mercury–cadmium-telluride (MCT), etc.). While compact “shoebox-sized” FTIR spectrometers are available, the push toward miniaturization toward absorbance-based MIR sensors has largely been driven by advancements in MIR semiconductor laser technology such as quantum and interband cascade lasers (QCLs, ICLs) and on-chip quantum-well photoconductive or quantum cascade detectors (QUIPs, QCDs). While the miniaturization of light sources and detectors has resulted in significant improvements, reducing, for example, the dimensions of the gas cell directly impacts the analytical sensitivity due to a decrease in absorption path length. To address this challenge, alternative waveguide technologies such as photonic bandgap waveguides and integrated hollow waveguides for the gas-phase samples and thin-film semiconductor waveguides for liquid/solid samples have been developed for achieving sensitivities at low parts-per-million (ppm) to parts-per-billion (ppb) levels.^22,23^

The following sections will discuss the most recent technological advancements related to key MIR components including light sources, waveguides, and detectors with a specific focus on progress toward device miniaturization.

## RECENT ADVANCES IN KEY MID-INFRARED COMPONENTS

### Mid-Infrared Light Sources

#### Interband Cascade Lasers (ICLs)

Interband cascade lasers (ICLs) are light sources operating in the MIR range that combine the electron–hole recombination process of conventional semiconductor lasers with the cascading architecture found in quantum cascade lasers (QCLs). This hybrid design allows for efficient operation with lower voltage requirements and improved energy utilization. However, early designs faced challenges, such as high threshold current densities and suboptimal performance at elevated temperatures. Recent advancements led to significant improvements in several critical areas including but not limited to efficiency, emission wavelength range, and operational temperature.^24^

One of the key developments in recent years has been the extension of the operational wavelength range of the ICLs. The continuous-wave (CW) operation of ICLs beyond 12 μm has been reported utilizing InAs_0.5_P_0.5_ barriers in the quantum wells of the active region. This innovation allows for longer wavelength operation by lowering the electronic energy level reducing the InAs well width, and enabling the emission of light at longer wavelengths.^25^ Furthermore, Shen et al.^26^ demonstrated ICLs that operate at wavelengths up to 14.4 μm, which sets a record for III–V interband lasers. This achievement suggests that ICLs could be further evolved to cover an even broader wavelength regime, offering enhanced versatility for applications in the long-wave region of the MIR.

Another major advance has been achieved with InAs-based ICLs. Huang et al.^27^ demonstrated continuous-wave (CW) operation at a maximum temperature of 41 °C, representing a 66 °C improvement vs previous devices operating near 5.2 μm.^28^ Additionally, the reported threshold current density of 306 A/cm^2^ during pulsed operation at room temperature is the lowest value recorded for semiconductor lasers at similar wavelengths.

Finally, adjustments of the hole injector have notably improved the carrier transport within ICLs. This enhancement is largely attributed to changes in the composition of the GaInSb well, which minimizes strain buildup and lowers the defect density resulting in improved material quality and overall device performance.^29^ Moreover, the CW output power has now reached 10 mW at 20 °C. Although this is still lower vs some GaSb-based ICLs, a major step forward in optimizing InAs-based ICLs for MIR applications has been achieved.^29^

#### Quantum Cascade Lasers (QCLs)

Quantum cascade lasers (QCLs) are unipolar semiconductor devices that operate via electronic transitions within a single conduction or valence band.^30^ Significant progress has been made using material systems such as InGaAs/AlInAs on InP and GaAs/AlGaAs supporting room-temperature operation and CW operation.^31^ Further developments in material systems like AlAsSb and III-nitrides aim to extend the emission to the shorter wavelength regime, although challenges in growth and processing remain to be resolved. Moreover, advancements in growth techniques such as molecular beam epitaxy (MBE) and metal–organic chemical vapor deposition (MOCVD) have enabled an even more precise control on the deposited atomic layers resulting in improved QCL efficiency broadening their utility for MIR sensing scenarios.^32^

Recent progress in multiwavelength QCLs has focused on improving integration and miniaturization for on-chip MIR sensing systems. A significant development is the hybrid integration of dual distributed feedback (DFB) QCLs onto a silicon photonics platform using a 3D self-aligned flip-chip assembly for precise alignment with submicrometer accuracy. This Ge-on-Si platform provides low optical losses and compatibility with CMOS processes, achieving an optimal coupling efficiency of 61.8% between the QCL and the Ge waveguide. This has enabled QCLs to operate at room temperature with a threshold current of 170 mA, submilliwatt optical output, and a possibly compact footprint advancing portable high-performance MIR devices.^33^

Extending the utility of QCLs into the far-IR (e.g., terahertz) regime, significant efforts have focused on increasing their operating temperature currently exceeding 200 K. Enhancements in injection barrier and active region design have improved carrier transport control, have reduced thermal backfilling, and have enabled stable operation at higher temperatures without compromising the performance.^34^ Further progress has been made in single-mode QCL operation across the 3.8–8.3 μm range. Innovations include antireflective (AR) coatings at both facets and improved side-mode suppression for enhanced single-wavelength output. Optimized QCLs now achieve up to 5.1 W light output power and 16.6% wall-plug efficiency, rendering them ideal for applications such as long-range free-space communication and remote sensing of hazardous chemicals, as needed for, for example, gas leak detection in industrial environments.

Further developments have been made in the design of QCLs by incorporating an extended ridge width, which reduces mode loss, enhances beam quality, and enables higher output power. The use of diamond heat sinks improves the thermal management and contributes to a higher maximum output vs previous designs. A step taper of the active region has also been implemented to enhance internal quantum efficiency, leading to more efficient carrier transport. These innovations resulted in an output power of 2.2 W at 5 μm with an 8.0% wall-plug efficiency. However, increasing the ridge width leads to higher temperatures in the active region, which may affect thermal efficiency and necessitate more effective heat dissipation management. Table 1 summarizes the performance parameters of recently developed QCLs and ICLs.

**Table 1:** Summary of the Performance Parameters of Contemporary QCLs and ICLs

laser source	emission wavelength	threshold current density	output power	maximum operating temperature	ref
ICL	10–13 μm	12–26.7 A/cm^2^ (80K)	32 - 56 mW	123 K (continuous wave mode) 160 K (pulse mode)	(25)
ICL	14.4 μm	13 A/cm^2^ (80K)	-	212 K	(26)
ICL	5.17 μm	306 A/cm^2^	10 mW	314 K	(27)
ICL	10–12 μm	8.8 A/cm^2^ (80K)	109 mW	137 - 187 K	(29)
ICL	3.25–3.42 μm	40 mA	42 mW (GaSb)	341 K (GaSb)	(35)
ICL	3.25–3.42 μm	40 mA	37 mW (GaAs)	338 K (GaAs)	(35)
ICL	3.25–3.42 μm	40 mA	32 mW (Si)	336 K (Si)	(35)
					
QCL	7.2 μm	170 mA	0.7 mW	228.15 K	(33)
QCL	3.8 μm	2550 A/cm^2^	1600 mW	–	(36)
QCL	4.9 μm	1920 A/cm^2^	5100 mW	–	(36)
QCL	8.3 μm	1060 A/cm^2^	3400 mW	–	(36)
QCL	5 μm	970 A/cm^2^	2200 mW	288 K	(37)

#### Light-Emitting Diodes (LEDs)

LEDs are semiconductor devices that emit light if an external voltage is applied, driving charge carriers into the active region where they recombine to produce light. Similar to traditional LEDs, MIR LEDs work by injecting charge carriers into an active layer, where they recombine radiatively to produce light. However, to efficiently emit light in the MIR range, such LEDs frequently require specialized materials and designs. These devices typically feature an active layer made from narrow-band gap semiconductors such as InAs or GaSb sandwiched between charge-transporting layers (CTLs). The CTLs aid in injecting and transporting electrons and holes while preventing unwanted recombination at the electrode interface, thus improving the overall device efficiency.^32,38^

Recent advancements in MIR LEDs have led to significant improvements in light output, radiance, and energy efficiency. By incorporating a W-superlattice structure and surface texturing, these LEDs exhibit a 2-fold increase in radiance compared to earlier designs, especially at cryogenic temperatures.^39^ At room temperature, these LEDs show three-times brighter output vs previous 4.2 μm LEDs. Additionally, these devices consume 100 times less energy vs conventional thermal emitters used in gas sensing, thereby enhancing the energy efficiency of MIR sensors.

Building on these improvements, a recent study has presented MIR LEDs with a simplified structure that uses asymmetric gold and graphene contacts, reducing the complexity typically associated with 2D light-emitting devices. This innovation not only makes the manufacturing process more cost-effective but also enhances the carrier injection efficiency. By combining the narrow bandgap of black phosphorus with the adjustable Fermi energy of graphene, the device facilitates simultaneous electron and hole injection for improved performance characteristics.^40^ This study by Zhang et al. also explores hot carrier injection and band filling at high current conditions, providing valuable insight for future device optimization. These advancements suggest the scalability of the design, rendering LED-based light sources well-suited for optical communications and gas sensing applications.

The development of MIR HgTe-based colloidal quantum dot LEDs has made significant advances, particularly related to the emission efficiency at 4 μm, which remains a challenge due to slower rates of the radiative processes vs the faster nonradiative processes. These devices achieved an external quantum efficiency of 10^–3^ and a power conversion efficiency of 10^–4^ with performance improvements driven by innovations in electrode design.^41^ Specifically, the use of a metal conductive grid to reduce the transparent electrode resistance from 150 Ω to 10 Ω was a notable advancement, enabling higher electroluminescence intensity at lower bias. The study also explored the effects of temperature and bias, revealing how thermal carriers and electron–hole recombination efficiency impact the device performance. While the device architecture was not the primary limiting factor, the material properties and fabrication techniques were found to play a more critical role.

Overall, these advancements highlight the potential for improving MIR LEDs despite challenges in thermal management and photoluminescence efficiency, suggesting that future developments will focus on optimizing material properties and thermal management to overcome these limitations. Recent advancements in silicon-based MIR optoelectronics have demonstrated notable progress in the integration of high-performance light-emitting devices onto silicon substrates. Studies indicate that superlattice light-emitting diodes (SLEDs) grown on silicon outperform those on GaSb substrates, particularly in large-scale devices operating at high injection levels.^42^ These performance gains are largely attributed to the superior optical transparency of silicon and the thermal conductivity, which enable efficient operation at demanding conditions, such as increased radiance due to lower substrate absorption, improved thermal management that prevents overheating under high injection conditions, reduced sensitivity to duty cycle changes, and enhanced performance in larger devices operating in high injection.^42,43^ Innovations such as migration-enhanced epitaxy and strained layer dislocation filters have mitigated lattice mismatch challenges, improving the structural quality and reducing defects. These advancements position silicon as a scalable and cost-effective substrate for advanced MIR optoelectronic devices.

Adding to this progress, Delli et al. have shown the successfully integration of InAsSb p-i-n LEDs onto silicon wafers using a GaSb buffer layer, enabling the growth of high-quality crystalline InAsSb layers.^44^ Thereby, electroluminescence at room temperature has been achieved with an external quantum efficiency of 0.011% and an output power of 6 μW at a 190 mA drive current. The LED structure consisted of a 2-μm-thick *n*-type InAsSb layer, a 1 μm undoped InAsSb emission layer, a 50 nm undoped AlAsSb electron-blocking barrier, and a 500-nm-thick *p*-type InAsSb contact layer. The use of the AlAsSb barrier enhances the conduction band offset, improving electron confinement and efficiency.

#### Supercontinuum Laser Sources

Supercontinuum sources have emerged as a transformative technology, expanding laser spectra through nonlinear optical processes and opening new possibilities in biophotonics, diagnostics, and infrared imaging. These sources offer MIR capabilities not accessible by traditional light sources such as QCLs. Despite challenges like spectral instability and long-term performance issues, recent advancements have significantly enhanced spectral bandwidth, power output, and noise performance.^45,46^

In particular, fluoride fibers, such as those made from InF_3_, have shown promise in providing wide MIR coverage. Using a high-peak–power femtosecond mode-locked fiber laser as the pump source, these systems can generate supercontinuum radiation that spans from 1.25 μm to 4.6 μm, with suitable spectral coherence. Advances in fiber geometry, material selection, and system architectures have enabled these sources to deliver bright, high-power output for gas sensing, chemical analysis, and infrared spectroscopy.^45^

To enhance noise performance of supercontinuum sources, a recent method introduced a soliton-based supercontinuum (SC) source involves incorporating a short segment of normal dispersion fiber. This technique forces solitons to broaden via self-phase modulation and optical wave breaking, effectively averaging out the noise. The method was experimentally and numerically validated using a ZBLAN fiber-based MIR SC source, with a highly nonlinear arsenic-sulfide (As–S) fiber enhancing the noise reduction.^47^ Simulations suggest that increasing the spectral broadening in the As–S fiber could further improve noise reduction, leading to even more refined and detailed images in the OCT, thus improving its diagnostic capabilities and performance in various applications.

Moreover, current demands have led to advancements in both materials and fiber design. An innovation is the use of photonic crystal fibers (PCFs) made from Al_0.24_Ga_0.76_As, a semiconductor known for its high nonlinearity that enhances the supercontinuum generation process in the MIR range. Adjusting the size of the air vents in the cladding has allowed researchers to achieve a zero-dispersion wavelength (ZDW) of 3.1 μm, optimizing the efficiency of supercontinuum sources and expanding their potential for broadband light generation in applications like chemical sensing and spectroscopic analysis.^48^

Expanding on these advancements, all-solid hybrid microstructured optical fibers (ASHMOF) have proven to be a more efficient platform for MIR supercontinuum generation due to their precise tailored dispersion properties, which enhance high-power supercontinuum generation. These fibers are designed with an optimized core-cladding structure and nonlinear materials, such as germanium and tellurite glass, to improve spectral broadening. Compared to traditional fiber-based sources, ASHMOF fibers offer wider spectral coverage and better coherence, making them suitable for high-precision applications, such as gas sensing, chemical analysis, and environmental monitoring, while overcoming the limitations of earlier technologies.^49^

## WAVEGUIDES

### Planar Waveguides

Evanescent field spectroscopy has undoubtedly become the leading miniaturized optical technique for liquid-phase IR sensing—particularly for water-based samples—as the sampled volume is determined by the penetration depth of the evanescent field, typically only a few micrometers at MIR wavelengths.^13^ As previously discussed, the number of total internal reflections within the waveguide depends on its thickness, structure, and geometry, which directly impact the sensitivity of the analytical method. Initially, single-reflection bulky internal reflection elements were used in ATR-based spectroscopy. These systems often suffered from low sensitivity and were primarily applied for qualitative analysis. Subsequently, optical fibers emerged as sensing components, offering a viable alternative to traditional IREs. In these waveguides, the orientation of active zones can be optimized to enhance internal reflections, particularly when micrometer-scale fibers. Although optical fibers improved sensitivity, their integration into “on-chip IR sensing” systems remains limited, due to challenges related to mechanical strength.

**Figure 1:**
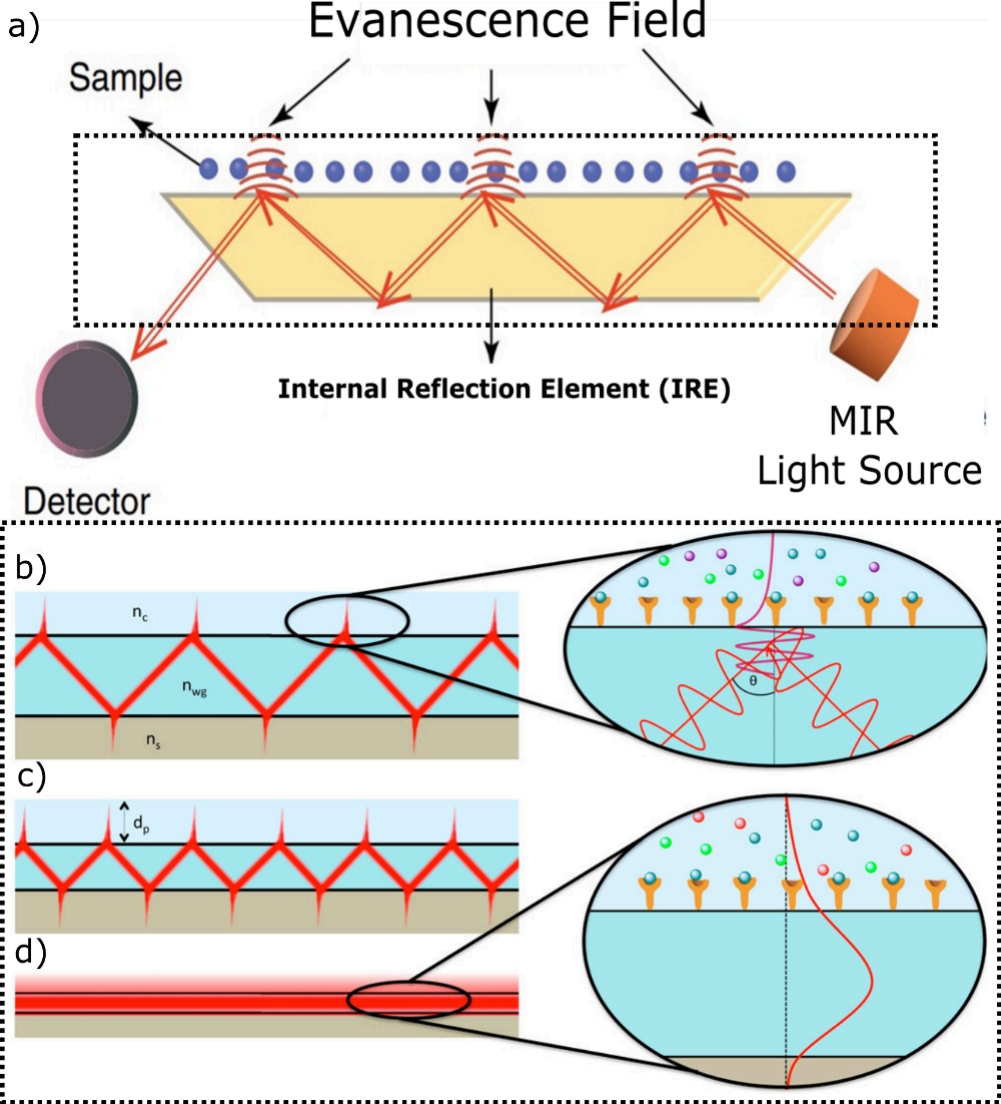
(a) Representation of a typical setup of an ATR-based spectrometer. Panels (b–d) shows schematics of a planar waveguide composed of a layer of sample (e.g., analyte-containing solution, solid, etc.) (nc), a waveguiding layer (nwg), and a substrate layer (ns) (i.e., in case of a thin-film substrate-supported waveguide structure) with nwg > ns, nc: (b) the guided radiation propagates via total internal reflections along a zigzag path through the waveguide; (c) as the thickness of the waveguide decreases, the number of internal reflections increases; and (d) if the thickness is on the order of magnitude of the wavelength, individual reflections no longer adequately describe the propagation behavior of the wavefront. The evanescent field appears continuously along the entire waveguide surface. [Reproduced from ref (9). Copyright 2016, American Chemical Society, Washington, DC.]

In this scenario, planar waveguides—also referred to as the third generation of waveguides—offer several distinct advantages over bulky traditional IREs. These include a miniaturized design, efficient on-chip integration, low sample volume requirements, high sensitivity, and straightforward operation.^9,50^ The thickness of most traditionally used waveguides is significantly larger than the wavelength in the MIR region, limiting the number of total internal reflections and reducing the effective interaction with the sample. Figure 1a represents a typical IR-ATR spectroscopy setup, which essentially also reflects the key components for MIR sensing systems and the dependence of TIR on the thickness of a planar waveguide and its impact on the depth of the evanescent field. In the configurations shown in Figures 1b and 1c, the number of reflections increases as the waveguide thickness decreases. When the thickness approaches the order of magnitude of the wavelength, individual reflections no longer adequately describe light propagation, as depicted in Figure 1d. In this type of waveguide—referred to as an integrated optical waveguide (IOW)—the evanescent field is continuously generated along the entire waveguide surface. The propagation of light in such structures is described by solving Maxwell’s full-wave equations. Briefly, wave propagation can be categorized as either single-mode (where only fundamental modes propagate within the structure) or multimode, depending on the waveguide thickness.

**Figure 2:**
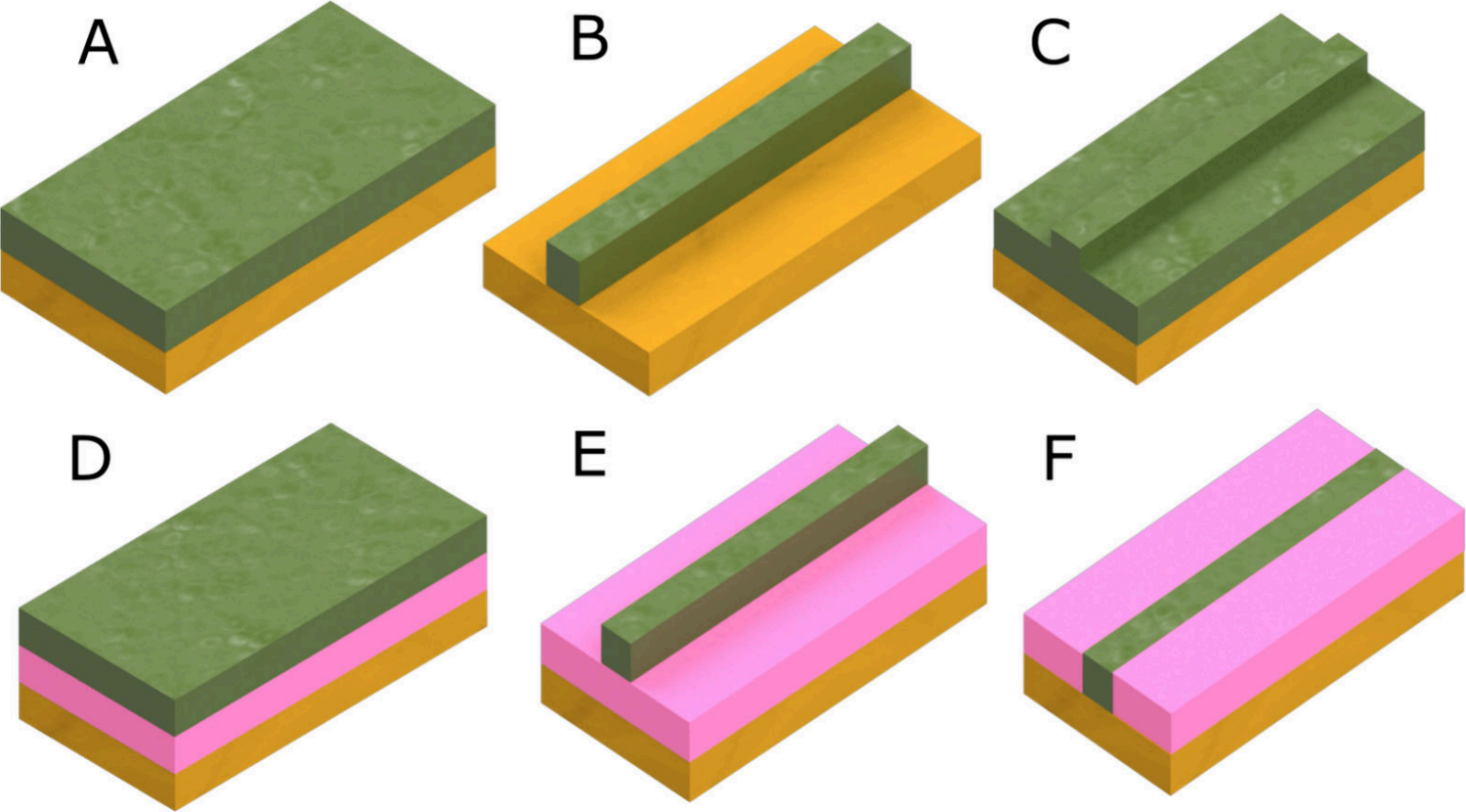
Illustration of common thin-film waveguide geometries: (a) slab waveguide, (b) strip waveguide, (c) rib waveguide, (d) slab waveguide, (e) ridge waveguide, and (f) embedded/buried waveguide. The same functional layers are marked with the same colors: green = waveguide layer (nc), pink = optical buffer layer (nb), and yellow = substrate (ns). [Adapted from ref (50). Copyright 2022, MDPI.]

The geometry of thin-film planar (i.e., slab) waveguides, in addition to their thickness, significantly influences wave propagation. Propagation occurs not only vertically along the long axis, as illustrated in Figure 1, but also laterally within the waveguide layer, potentially enhancing optical strength. Consequently, various geometric designs for thin-film waveguides have been developed, enabling both single-mode and multimode light propagation. The most common geometries used for waveguiding are schematically illustrated in Figure 2. Typically, a waveguide system consists of layers of a waveguide, an optical buffer, and a substrate. These layers are created through deposition, etching, or annealing processes using materials with different refractive index. This combination facilitates the fabrication of efficient thin-film waveguide systems for the MIR range. A detailed discussion of the differences between these geometries is beyond the scope of this Review but can be found in numerous comprehensive studies available in the literature.^9,50,51^

Lastly, the choice of waveguide material defines the application range of the IR sensor, as transparency within the spectral region is a critical factor. Additionally, mechanical robustness, durability, high refractive index, chemical inertness, and biocompatibility are essential considerations during the fabrication and application of ATR waveguides. Planar waveguides made from chalcogenides (e.g., S, Se, As, and Te) have emerged as exceptional materials for thin-film MIR devices due to their broad optical transparency (i.e., 2–10 μm) and high refractive index (i.e., >2).^51^ Combining chalcogenides with elements such as As, Ge, and Ga extends the MIR spectral window to wavelengths of up to 20 μm. Other materials have also been utilized in manufacturing thin-film waveguides, and their key optical parameters and important remarks were summarized in a previous review.^9^ Most materials are transparent only within limited spectral ranges, with notable exceptions such as HgCdTe, CdTe, and diamond, which exhibit transparency across the MIR region. Conversely, SiO_2_, commonly used in visible and NIR applications, is often employed as a cladding layer to establish more sophisticated—and complex—waveguide designs. In the following sections, we provide a more-detailed review of waveguide fabrication using different materials.

#### Chalcogenide Waveguides

High-quality optical glasses with properties such as a high refractive index, broad infrared transparency, low phonon energy, and high optical nonlinearity—along with stable optical performance— are essential for miniaturized planar waveguide-based devices. Chalcogenide glasses, typically composed of S, Te, and Se, combined with elements like Ge, As, and Ga, have been extensively studied over the past 70 years due to their versatile applications in fields such as optoelectronic devices.^52,53^ Their intrinsic high refractive index, glassy structure, low material loss, and compatibility with various deposition techniques make amorphous chalcogenide films a favorable choice for integration into “on-chip” IR platforms.

Despite their well-established applicability, chalcogenide glass-based waveguides, used as cladding or waveguide layers, face limitations due to attenuation losses starting from 10 μm. To expand the transmission wavelength range for analytical applications, we have developed waveguides made with composites containing germanium, tellurium, and gallium have been developed. Low-loss single-mode GeSbSe waveguides were designed and fabricated using a combination of thermal evaporation, electron beam lithography (e-beam lithography, EBL), reactive ion etching (RIE), and inductively coupled plasma etching (ICP).^54^ To prevent the formation of bubbles, GeSbSe films were thermally evaporated at 10^–7^ Torr, starting with an initial deposition rate of 1 Å/s, which was then increased to 10 Å/s after 100 nm. MgF_2_ substrates, with a thickness of 0.5 mm and a surface roughness of 0.5 nm, were used, and the films were annealed at 265 °C under a nitrogen atmosphere to promote adhesion between the chalcogenide films and the substrate. Cytop 809 M was used as the cladding layer. Etching with CHF_3_ resulted in 1.5 dB/cm higher propagation losses at 3.6 μm, compared to BCl_3_. Finally, the GeSbSe waveguide, cladded with 50 nm of Cytop to prevent the adsorption of chemical compounds onto the chalcogenide surface, was fabricated with dimensions of 1.2 μm × 0.88 μm. This waveguide was used to determine the concentration of aromatic formaldehyde (i.e., cinnamaldehyde) by measuring the loss of transmitted light at 3.664 μm.

As_2_Se_3_ waveguides are transparent in the MIR region due to their low phonon energy. Using microelectronic fabrication processes, ridge As_2_Se_3_ waveguides were created on SiO_2_ undercladding via sputtering and lift-off techniques, rather than chemical etching or thermal annealing. These waveguides were capable of integrating with wireless microelectronics to form a sensing device for VOC quantification.^55^ The significant refractive index contrast between As_2_Se_3_ (2.79) and SiO_2_ (1.45) allowed for the confinement of MIR light within the waveguide core. A 0.16 dB/cm loss was measured at 3.5 μm, demonstrating that the sputtered chalcogenide glass is highly transparent in the MIR range. Strong mode intensity was observed between λ = 3.40 and 3.60 μm. Upon injecting acetone and ethanol vapor over the waveguide, the mode intensity sharply decreased at λ = 3.40–3.42 and 3.40–3.52 μm, respectively. The bending loss of a channel optical waveguide with an As_2_Se_3_ core and an AsS_3_ cladding layer was theoretically evaluated within the MIR region (8–12 μm). It was found that this loss can be reduced by increasing the waveguide thickness (*w*_c_) and the change in refractive index (Δ*n*).

Although some chalcogenide films exhibit high optical transparency and a high refractive index, their optical stability tends to be slightly limited. The effect of thermal annealing on the optical stability of Sb_2_Se_3_ thin-film waveguides doped with GeTe, prepared by sputtering, was evaluated in terms of optical and thermal properties.^56^ The study found that optical and thermal stability, as measured by changes in the bandgap and refractive index, was optimized when GeTe was present at 15.7%. An interesting paper by Lin et al. described the integration of an interband cascade laser (ICL) emitting at 3.24 μm with a passive, high-index contrast waveguide made of chalcogenide glasses.^53^ The on-chip structure consisted of a 3 mm ICL and a 1.5 mm waveguide. The multilayer on-chip structure was fabricated on an *n*-GaSb substrate and included an InAs-AlSb superlattice (SL) optical cladding layer. The chalcogenide waveguide featured an As_2_Se_3_ (*n* = 2.78) core, sandwiched between Ge_23_Sb_7_S_70_ (*n* = 2.13) top and bottom claddings. This approach marks a significant step toward integrated MIR photonic circuits.

#### Silicon Waveguides

Silicon-based photonics has emerged as one of the most competitive solutions for integrated sensing systems in the visible and near-IR (NIR) spectral ranges. Among these, silicon-on-insulator (SOI) and silica-on-silicon are the most prominent platforms for the MIR range.^57^ However, the strong absorption of SiO_2_ in the 2.6–2.9 μm range and beyond 3.6 μm, as well as sapphire’s absorption at wavelengths above 6 μm, limits their applicability.^9^ Additionally, monocrystalline silicon offers optical transparency of only up to 8 μm. To extend the effective application window, multilayer waveguides have been developed, such as germanium-based silicon (Ge-on-Si),^17^ silicon carbide (SiC),^58^ SiGe grade index on silicon (SiGe-on-Si),^59^ and silicon-on-nitride (SiN)^60^ platforms. However, the challenging fabrication strategies involved, including suspended multiple thin-film growth and selective etching steps, have hindered device development and limited their widespread application.

Femtosecond laser inscription enables the creation of microfluidic channels and optical waveguides within a fused silica substrate.^61^ When fused silica is exposed to tightly focused femtosecond laser pulses, the laser beam induces a permanent localized modification of the glass’s optical and chemical properties within the focal volume. The fabricated switch exhibits a total internal reflection loss of approximately 1.5 dB at a wavelength of 1.5 μm. A multistrip integrated waveguide for MIR sensing was fabricated using silicon substrates.^62^ Initially, a system plane of SiO_2_ (2 μm) and Si_3_N_4_ (140 nm) was deposited on the substrate to act as an insulating layer. Then, 660 nm of undoped polysilicon (Poly-Si) was deposited via chemical vapor deposition, followed by thermal treatment at 900 °C and photolithography with dry etching. An AlN and metallic layer were then deposited to form the final waveguide structure, with a strip width of 1.3 μm and a gap width of 0.4 μm. This structure has the potential for integration into sensor systems with low intrinsic damping across various processes. In addition to waveguides, integrated Si and Si_3_N_4_ photonics have shown significant promise for performing multiple optical functions, such as light generation, guidance, and detection, laying the foundation for on-chip integrated optical fabrication. Furthermore, a large-area homogeneous monolayer of WS_2_ was directly grown onto a Si_3_N_4_ waveguide via controlled physical vapor deposition using a SiO_2_/Si wafer as the substrate.^61^ The waveguide pattern was created by using electron beam lithography and ion etching, and an enhancement of second-harmonic generation was observed.

SiN thin films, produced via low-pressure chemical vapor deposition (LPCVD), exhibit a wide MIR transparency range and a lower refractive index (*n*SiN = 2.0) compared to conventional materials like silicon (*n*Si = 3.4).^63^ This lower refractive index enhances the evanescent field, resulting in a greater sensitivity. As previously mentioned, the fabrication of Si-based waveguides typically involves multiple SiN/SiO_2_ layers. The number of layers, along with their width and thickness, influences both the mechanical strength and the optical properties of the waveguide. The impact of the geometry and number of layers was evaluated through simulations to optimize waveguiding at 2.64 μm. Losses below 6 dB were measured for a waveguide made of 125-nm-thick SiN layers and 90-nm-thick SiO_2_ layers, indicating near-perfect waveguiding performance.^64^

A Si-on-SOI waveguide circuit integrated with a PDMS microfluidic channel was fabricated, featuring a pair of optical switches that alternated light between an evanescent absorption sensor waveguide and a reference waveguide at a frequency of a few kHz.^65^ This design achieved an 11-fold reduction in the noise floor compared with a standard waveguide absorption sensor. The device’s performance was validated by detecting an isopropyl alcohol (IPA) absorption peak at a wavelength of 3.77 μm.

Lastly, silicon carbide (SiC) is gaining recognition as a promising material for integrated quantum photonics due to its unique properties, including a high refractive index, strong second- and third-order optical nonlinearities (arising from its wide bandgap and suppressed two-photon absorption at telecom wavelengths), and a broad transparency window spanning from the visible to the MIR range. In a recent study,^66^ amorphous SiC (a-SiC) films were deposited using inductively coupled plasma chemical vapor deposition (ICP-CVD) onto 2.5 μm thermally grown SiO_2_. The film thicknesses were selected based on finite-difference time-domain (FDTD) simulations to ensure single-mode waveguide operation, resulting in thicknesses of 0.28 and 0.75 μm.

#### Diamond Waveguides

Among Group IV materials, silicon has long been the primary choice for photonics applications, because of its ubiquity and availability.^67^ However, as mentioned previously, its transparency in the MIR spectrum is typically limited to 8 μm. In this context, diamond, a less-utilized member of the group, has emerged as an excellent alternative.^68^ Diamond offers exceptional optical transparency across the entire MIR range (with the exception of two phonon absorption peaks between 4 and 6 μm), chemical inertness, superior thermal and mechanical properties, and a high refractive index (*n* = 2.421 at 6 μm).^69^

Traditionally, diamond has been used as a single internal reflection element in ATR spectroscopy. This application was driven by the limited power of standard MIR light sources and the challenges of reducing the dimensions of bulky diamond crystals. Recently, advancements in fabrication techniques have enabled the production of thin-film diamond waveguides using single-crystalline (SCD) or polycrystalline (PCD) diamond with thicknesses comparable to MIR wavelengths (e.g., 16 μm).^70−72^ Combined with the development of more powerful light sources, these thin-film diamond waveguides have facilitated the quantification of various molecules by MIR spectroscopy. While SCD-based materials deliver high performance, their fabrication costs are significantly higher than those of PCD, despite comparable optical performance in the MIR range. Conventional cladding materials, such as SiO_2_, absorb strongly at longer wavelengths, making them unsuitable for integration with diamond. To address this, free-standing suspended waveguides with air cladding supported by a silicon frame have been explored. However, challenges such as high strain in the diamond layers, thermal expansion mismatches, and stringent high-temperature processing requirements complicate fabrication.^9^ An alternative approach involves growing thin-film diamond on silicon substrates and partially removing the silicon layer to achieve stable growth conditions and refractive index compatibility. While free-standing diamond structures are self-supporting,^14^ their inherent brittleness makes them mechanically fragile, a limitation not encountered with bulk diamond IREs.

Nanocrystalline diamond thin films grown directly on silicon substrates have been studied and designed as air-clad suspended ridge waveguides for operation across the 2.5–16 μm spectral range. TFWGs with thicknesses of 520, 1000, and 2000 nm were used to target three spectral regions: 2.5–5 μm (520 nm), 4–9 μm (1000 nm), and 8–16 μm (2000 nm).^73^ Despite the low thickness-to-wavelength ratio, these waveguides demonstrated mechanical stability, effective optical confinement, and low bending losses (below 10 dB beyond 6 μm). Ridge diamond waveguides incorporating aluminum nitride (AlN) cladding have been fabricated by using various methods. Since direct diamond deposition on AlN is time-intensive, an alternative process was developed: a 5-μm-thick PCD film was deposited on a silicon wafer via chemical vapor deposition (CVD) and polished.^14^ The wafer was laser-cut into 5 mm × 10 mm samples, and AlN was sputtered onto the diamond layer in four 30-min cycles, with cooling periods to prevent cracking or peeling. The silicon substrate was subsequently removed by dry ICP-etching using argon and sulfur hexafluoride. The resulting waveguides, employing AlN cladding, operated effectively in the 7.7–13.7 μm range, outperforming SiO_2_-clad waveguides. This represented the first experimental demonstration of a diamond-on-AlN waveguide for MIR spectroscopy, with improved robustness and enhanced sensitivity due to the thinner diamond layer.

Collaborative efforts between the research groups of Mizaikoff and Karlsson have significantly advanced diamond waveguides for integrated IR sensing. A polycrystalline diamond trapezoid IRE grown by CVD was coupled with a tunable quantum cascade laser, achieving a 5-cm optical path length and enabling sensitive ATR measurements.^68^ A free-standing waveguide with a 16-μm-thick diamond layer was grown atop a 0.2-μm Si_3_N_4_ layer and a 2-μm SiO_2_ layer. Photolithography was used to create three mask layers – 1.7-μm aluminum, 0.8-μm silicon, and 0.13-μm aluminum – and ICP-etching and sputtering were performed to produce waveguides with widths of 14 and 500 μm. A recent study systematically compared waveguide designs for on-chip spectroscopy devices, evaluating GaAs, Se_6_/Se_2_, Ge-on-Si, and PCD waveguides at 6 and 10 μm.^74^

Additionally, metallic nanostructures added onto the waveguide may act as signal amplifiers to achieve pronounced electromagnetic near-field confinement and associated enhancement effects, thereby improving the analytical sensitivity. This approach is termed SEIRA (surface-enhanced infrared absorption) spectroscopy, and recently, graphene has been introduced as a surface-enhancing material owing to its unique properties, which have led to the introduction of so-called GEIRA (graphene-enhanced infrared absorption) spectroscopy. Diamond thin-film waveguides were augmented by drop-casted graphene enabling surface-enhanced infrared absorption spectroscopy combined with quantum cascade lasers.^75^ The setup enabled the quantification of low levels of taurine as a model analyte.

In summary, the exceptional optical, thermal, and mechanical properties, combined with its chemical inertness and biocompatibility, make diamond a highly suitable material for nanocrystalline and polycrystalline free-standing waveguides in miniaturized IR spectroscopy setups. However, the complexity of the multistep fabrication processes required to produce robust waveguides with the necessary optical quality continues to hinder the widespread adoption of diamond-based materials for on-chip sensing applications.

#### Germanium Waveguides

The fabrication of on-chip sensors based on photonic integrated circuits (PICs) is facilitated by the compatibility of complementary metal-oxide semiconductor (CMOS) technology with appropriate optical materials. Among such materials, germanium-based platforms stand out as promising candidates due to their broad transparency up to 15 μm, high refractive index (∼4), and full CMOS compatibility. Various configurations of germanium-based PICs have been proposed, including germanium-on-silicon (Ge-on-Si, GOS),^76^ germanium-on-insulator (GOI),^77^ germanium-on-nitride (GON),^78^ and Ge-on-silicon-on-insulator (Ge-on-SOI).^79^

Ge-on-Si waveguides, in particular, offer an ease of fabrication, along with optical transparency in an analytically useful MIR range. For example, a Ge-on-Si slot waveguide with a 3-μm thick germanium layer and a 200 nm slot gap was fabricated using sputtering, e-beam lithography, evaporation, lift-off, and dry etching.^17^ At 4.2 μm, the waveguide exhibited propagation losses of 5.2 dB/cm for the channel waveguide and 4.8 dB/cm for the slot waveguide, which is attributed to the significant refractive index contrast between the Ge core and the surrounding low-index materials. Additionally, strip waveguides with a 20-μm width and 3-μm thick Ge-on-Si layers were fabricated using lithography and ICP-based fluorine etching chemistry.^76^ These waveguides were coupled to a tunable quantum cascade laser (QCL) operating from 5.3 to 1.9 μm and successfully detected bovine serum albumin (BSA) in the targeted spectral region.

Suspended germanium waveguides also show potential for light guiding in the MIR range. A suspended waveguide based on a Ge-on-SOI platform with a 50-nm silicon layer and a 3-μm-thick germanium core demonstrated propagation losses of 5.3 dB/cm at 7.7 μm.^80^ Moreover, the integration of Ge onto glass substrates was explored with other cladding materials. For instance, a germanium-on-zinc selenide (GOZ) waveguide, introduced by Burghoff’s group, exhibited low losses (1 dB/cm) at 7.8 μm and a transparency range extending from 2 to 14 μm.^81^ The fabrication process for the GOZ platform involves polishing, coating, lithography, dry etching, and dicing. These results position the GOZ platform as a versatile foundation for long-wave infrared (LWIR) integrated photonics, with adaptability approaching that of silicon-on-insulator (SOI) at shorter wavelengths. Germanium waveguides are widely utilized for MIR photonics due to their broad optical transparency in analytically significant spectral regions. The development of second-generation Ge-based waveguides (e.g., Ge-on-Si and Ge-on-ZnSe) has enabled the production of thin films with significantly improved light-guiding properties. These advancements have led to reduced propagation losses and expanded the potential for on-chip sensing applications in the MIR range.

#### GaAs/AlGaAs Waveguides

The success of silicon-based photonic integrated circuits over the past decade has prompted debate into whether similar fabrication techniques could be applied to materials with desirable optical properties in the MIR range. III–V semiconductor materials, such as gallium arsenide (GaAs) and aluminum gallium arsenide (AlGaAs), are well-established in electronic circuit design for their ability to support high-frequency data transfer. These materials can be precisely structured after epitaxial growth by using both wet and dry etching techniques. Advanced deposition methods, including metal–organic vapor phase epitaxy (MOVPE), physical vapor deposition (PVD), and molecular beam epitaxy (MBE), are commonly used to prepare thin-film semiconductors with high precision.^9^

GaAs emerges as a promising alternative to silicon for MIR optical applications due to its superior optical properties, including a direct bandgap, a weak piezoelectric coefficient, broadband low-loss operation, and a refractive index closely matching that of silicon. This compatibility enables the transfer of silicon photonic designs and fabrication techniques to GaAs-based platforms. AlGaAs, a widely studied III–V ternary semiconductor alloy, further enhances this versatility by allowing its properties to be modified through the aluminum concentration. Layers of AlGaAs can be grown on GaAs substrates with perfect lattice matching, regardless of the aluminum content.^1^

The research collaborative team led by Mizaikoff and collaborators pioneered waveguide-based sensors for the MIR spectral region using III–V semiconductor materials in the early 2000s and 2010s.^15,82^ Their work demonstrated that integrating photonic devices with common materials such as GaAs could significantly reduce the size of MIR analytical systems, enabling these materials to serve as light sources, detectors, and waveguide interfaces. Consequently, the implementation of PICs with suspended GaAs waveguides has been identified as a viable approach for developing miniaturized on-chip IR sensors.

Electron beam lithography for patterning and chemical etching for layer thickness control were employed to fabricate mechanically stable suspended rib waveguides composed of GaAs/AlGaAs.^83^ These waveguides featured a 540 nm-wide core and a total rib width of 6 μm. While maintaining the complexity of standard silicon photonics fabrication, transitioning to GaAs provides an enhanced optical performance. For example, a waveguide structure incorporating 100 repetitions of 8 nm GaAs/12 nm Al_0.32_Ga_0.68_As quantum wells as the active region was grown on a 2-μm-thick Al_0.7_Ga_0.3_As lower cladding layer, with a 2-μm-thick upper cladding.^84^ Transverse optical confinement was achieved by etching a 3-μm-wide ridge through the active region and lower cladding. The device was then cleaved into 3.6-mm-long samples.

AlGaAs heterogeneously integrated on silicon have attracted significant attention in recent years. The AlGaAs-on-insulator platform is notable for its exceptional nonlinear conversion efficiency, achieved by combining the high nonlinearity of the semiconductor with strong mode confinement enabled by the significant refractive index contrast with silica. For example, an AlGaAs layer grown via MBE on an oriented GaAs substrate was later bonded to a silicon wafer with a 3-μm-thick layer of thermal silica (SiO_2_). Recent studies have explored the integration of GaAs waveguides on various substrates. Moreover, GaAs-based materials offer versatile surfaces that can be tailored to specific biological applications. For instance, a biosensor was developed by functionalizing the surface of an undoped wafer composed of bulk GaAs/Al_0.35_Ga_0.65_As nanoheterostructures, which was subsequently employed in the MIR range. Finally, Table 2 summarizes recent studies using different types of waveguides for the MIR applications.

**Table 2:** Summary of Recent Published Papers Using Thin-Film Waveguides in the MIR Range

waveguide layer	propagation loss (dB/cm)	fabrication steps	application range	ref
GeSbSe	3.88	e-beam lithography/etching/thermal annealing	3.66 μm	(54)
As_2_Se_3_	0.16	lithography/sputtering	3.4–3.6 μm	(55)
GeSeTe	–	photolithography/etching/surface funcionalization		(85)
GeAsTeSe	–	thermal evaporation/ICP-etching/e-beam lithography/thermal deposition	2–13.2 μm	(86)
ChG/Ge	1.3/3	sputtering/photolithography/ICP-etching	4.3 and 7.7 μm	(87)
As_2_Se_3_/Ge_23_Se_7_S_70_	0.7	lithography/lift-off/evaporation	3.24 μm	(53)
Ge-on-Si	4.8	sputtering/e-beam lithography/evaporation/lift-off/dry etching	4.2 μm	(17)
Ge-on-Si	–	lithography/IPC etching	5.3–12.9 μm	(88)
Ge-on-Si	–	UV lithography/ion etching/dry etching	7.2 μm	(89)
Ge-on-SOI	5.3	e-beam lithography/ICP etching	7.7 μm	(80)
Ge-on-ZnSe	1	coating/lithography/dry etching	2–14 μm	(81)
GeSbSe	3.6	thermal evaporation/e-beam lithography/ICP etching		(54)
SiGe/Si	<1	chemical vapor deposition/UV lithography/ion etching	4 μm	(90)
a-Ge	–	chemical vapor deposition/sputtering/e-beam evaporation	3.5–7.5 μm	(91)
Native Ge	0.5	e-beam lithography/dry etching/lift-off/polishing	6–14 μm	(92)
Ge-SiC-Ge	–	chemical vapor deposition/ion etching	4.6 μm	(93)
GaAs-on-SOI	<7	MBE/chemical wet etchings	1 μm	(94)
AlGaAs-on-SOI	–	MBE	2.45 μm	(95)
GaAs-on-SOI	16.8	MBE/thermal bounding	2 μm	(96)
air-bridge AlGaAs	–	wet etching	1 μm	(97)

## HOLLOW WAVEGUIDES

### Photonic Bandgap Waveguides

**Figure 3:**
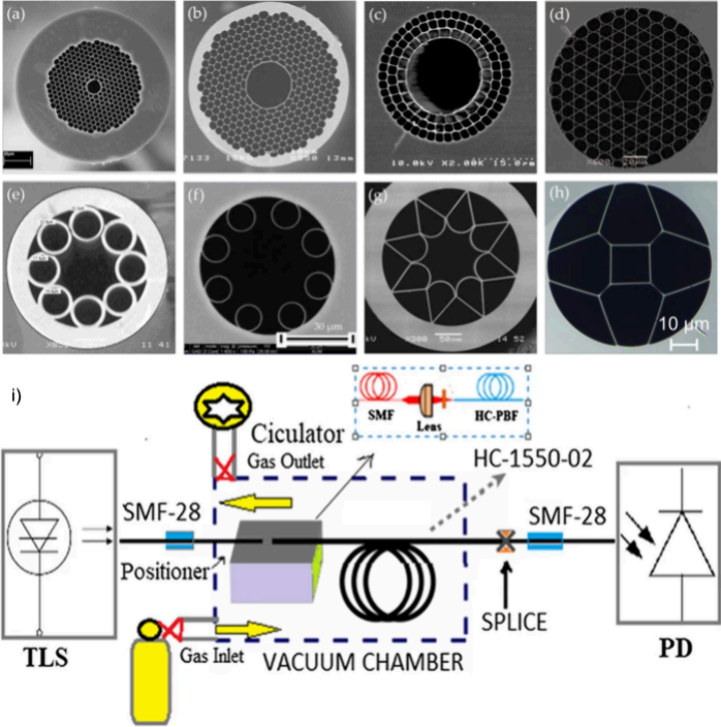
Panels (a–h) show scanning electronic microscopy (SEM) images of some representative hollow-core photonic crystal fibers (HC-PCFs). (a) Commercially available hollow core photonic bandgap fiber (HC-PBGF): HC-1550–02 was from NKT Photonics. [Reproduced (b) HC-PCF designed for the guiding in the MIR region; (c) hollow core Bragg fiber; (d) Kagome lattice HC-PCF; (e) hollow core antiresonant fibers (HC-ARF); (f) nodeless HC-ARF; (g) hollow fiber with negative curvature of the core wall]; and (h) double antiresonant hollow square core fiber. [Panel (a) was reproduced with permission from ref (101). Copyright 2020, MDPI.] (i) Schematic diagram of the proposed HC-PBGF based gas sensor. [Reprinted with permission from ref (100). Copyright 2021.]

A hollow-core photonic bandgap fiber (HC-PBF) enables the simultaneous confinement of light and gas- or liquid-phase samples within its hollow core, providing an excellent platform for strong light-matter interactions over extended distances.^98^ An HC-PBF features an air-filled core surrounded by microstructured glass cladding, enabling a high level of light confinement. Fused silica is the most commonly used material for these fibers, although polymers and soft glasses, such as chalcogenide and fluoride glasses, are also employed. Figure 3a illustrates a hollow-core photonic bandgap fiber (HC-PBGF) with a honeycomb lattice of air holes embedded in a silica background. The periodic lattice structure creates photonic band gaps. When the light frequency falls within the photonic bandgap, light propagation in the cladding is prohibited, confining it to the central air core, a low-refractive-index defect region. Figures 3a–h shows SEM images of representative HC-PBF structures.

Typically, the visible and NIR spectral ranges fall within the transmission band of hollow-core photonic bandgap fibers (HC-PBFs). However, single-mode operation for wavelengths exceeding 3 μm has already been demonstrated.^99^ Combining HC-PBFs with compact, miniaturized light sources (e.g., lasers) and detectors offers an excellent platform for developing on-chip IR sensing systems. In a typical setup, an adjustable light source is aligned to inject light into the HC-PBF via a single-mode fiber (SMF). At the output, a second SMF guides the transmitted light to a photodetector.^100^ The hollow core of the HC-PBF acts as a waveguide, facilitating strong light-gas interactions along the core due to the high overlap between the optical field and the sample (Figure 3i). This interaction between the light and optically active analytes attenuates the transmitted optical power due to absorption, enabling quantification via the Beer–Lambert law. The hollow-core radius and geometry were specifically optimized for detecting acetylene through MIR absorption.

A novel hollow-core negative curvature fiber (HC-NCF) was recently developed for CO detection via infrared laser absorption at 2.3 μm.^102^ The HC-NCF consists of a single ring of eight nontouching silica capillaries surrounding an air core, enabling single-mode light transmission from a 2.3-μm distributed feedback laser. This design achieved a high coupling efficiency of 90%, facilitated by optimized free-space coupling optics. The hollow-core fiber served as a gas cell, providing an effective absorption path length of 85 cm for the measurements.

Alternatively, additive waveguide fabrication through 3D nanoprinting using two-photon absorption-based direct laser writing of polymeric photoresists has demonstrated the creation of hollow-core light cage waveguides.^22^ These waveguides combine significant structural openness with low propagation losses. This innovative on-chip waveguide design enables strong light-analyte interactions and rapid response times while eliminating the need for complex multistep fabrication processes.

### Substrate-Integrated Hollow Waveguides

Building on the hollow waveguide concept while addressing the primary limitations of conventional HWGs, Mizaikoff and collaborators introduced a new generation of hollow waveguide structures known as substrate-integrated hollow waveguides (iHWGs).^23^ These feature a straightforward yet highly efficient modular design based on a layered structure. In this configuration, radiation is guided through an integrated straight or meandered light channel embedded within a solid-state substrate. Typically, iHWGs consist of two main components: (a) the base plate, which includes an integrated mirror-like hollow channel serving as both a gas cell and a reflective light guide, and (b) the top plate, which is mirror-polished to seal the channel and includes access ports for introducing gas samples. The two plates are joined and sealed using mechanical clamping devices, screws, or adhesives, such as epoxy. MIR-transparent windows (e.g., ZnSe, BaF_2_) are affixed to the waveguide channel facets at the radiation incoupling and outcoupling ends, ensuring efficient radiation transmission. A recent review on the development and applications of the iHWG concept can be seen in this Review.^7^

Significant advancements in iHWG manufacturing have been achieved through low-cost, time-efficient prototyping techniques like FDM and SLA-3D printing.^103,104^ Additionally, the deposition of thin-film waveguides inside the hollow channel of the iHWG enhances light guiding through the substrate, resulting in an extended optical path length and, consequently, lower detection limits. Recent studies also highlight the integration of metal-based iHWGs with surface-mounted device (SMD) interband cascade laser (ICL) LEDs emitting in the MIR region (i.e., 3.4–5.7 μm).^105^ These sensor systems have been tested as nondispersive infrared (NDIR) sensors in various scenarios, including the application of spectral filters, assessment of individual ICL-LED performance, and the analysis of gas mixtures.

**Figure 4:**
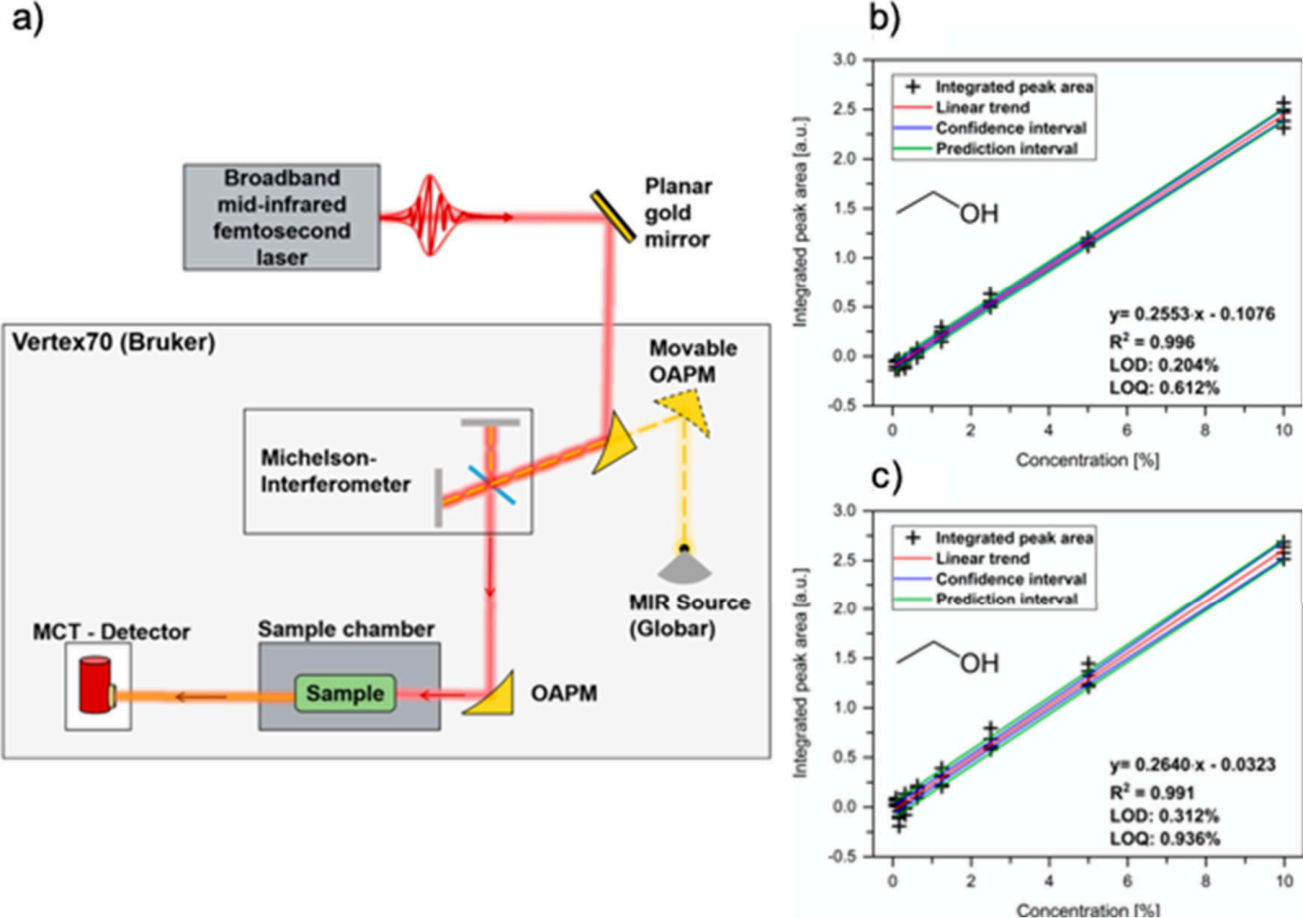
(a) Schematic illustration of the experimental setup for a broadband MIR femtosecond laser system (red line: laser source beam; yellow dashed line: globar radiation; OAPM: off-axis parabolic mirror; orange line: attenuated light after sample interaction MCT detector: mercury cadmium telluride detector). (b and c) Linear correlation between the integrated peak area and the concentration ranging from 0.08 to 10% of two independent ethanol measurements with their calculated linear regression equation, coefficient of determination (*R*^2^), and LOD and LOQ values. [Reproduced from ref (106). Copyright 2023, American Chemical Society, Washington, DC.]

Additionally, a hybrid analytical platform for liquid- and gas-phase samples was developed^106^ by integrating a tunable broadband MIR femtosecond laser source with two distinct components: a ZnSe crystal horizontal ATR sensor cell and a substrate-integrated hollow waveguide (Figure 4a). This platform enabled the quantitative analysis of three individual samples—ethanol (liquid), methane (gas), and 2-methyl-1-propene (gas)—achieving limits of detection of 0.3% for ethanol, 22 ppmv for methane, and 74 ppmv for isobutylene. These measurements were performed at specific emission wavelengths of the MIR laser source (11.2, 9.5, and 7.6 μm, respectively). Figures 4b and 4c present the linear correlation between the integrated peak area and the ethanol concentration.

Finally, a spectrally adaptable terahertz source, combined with iHWGs, provided a versatile platform for molecular sensing in the terahertz range. Its suitability for the terahertz domain was demonstrated by Theiner et al.,^107^ highlighting its low propagation losses and the successful measurement of rotational transitions of nitrous oxide (N_2_O). With continued advancements in miniaturizing all key components of optical chemical sensor systems, a wide range of future applications can be envisioned. These include ultralightweight, drone-mounted devices for environmental and agricultural monitoring, as well as disposable, low-cost sensor interfaces for medical and clinical applications, such as exhaled breath analysis in underserved regions.

## DETECTORS

Essentially, a photodetector converts the energy of the incident light into an electrical signal through devices fabricated with materials that can be roughly categorized into photon- and thermal-detection types.^108^ The ratio of the electrical signal output and the optical input as a function of monochromatic wavelength is defined as the photodetector spectral responsivity and is an essential figure-of-merit for several applications, including analytical chemistry.^109^ Moreover, the materials used in MIR detectors should offer characteristics such as large bandgap tunability, optical polarization sensitivity, and integrability with complementary materials (e.g., silicon) to provide on-chip and miniaturized devices.

Traditionally, commercial MIR detectors are made from conventional semiconductors with narrow bandgaps, such as HgCdTe (MCT), PbSe, and InGaAs.^110^ Despite its successfully application—especially MCT—with fast response and high efficiency, detectors made from these materials usually suffer from complex material synthesis, extremely low-temperature operation to prevent excessive heat during the excitation of carriers, and fixed band structure.^111^ Moreover, the integration with the complementary metal-oxide semiconductor (CMOS) process is challenging, which results in bulky terminal devices and, therefore, mitigating the potential for on-chip sensing systems.^112^

Pyroelectric photodetectors (PDD)—a type of thermal detector—have continuously been an attractive candidate due to their noncryogenic, low-cost, easiness of fabrication, bias-free and broadband characteristics.^113^ The classical pixel structure of a pyroelectric detector device (PDD) consists of two electrodes sandwiching a layer of pyroelectric material such as lithium tantalate (LiTaO_3_). In a recent study by Dao et al.,^114^ an ultranarrowband, on-chip multiwavelength MIR sensor was developed to operate across a spectral range of 3.522–3.922 μm. This multiwavelength detector was fabricated on a single silicon chip using a CMOS-compatible MEMS design. A metasurface-based pyroelectric detector was proposed, featuring a top electrode constructed with an absorptive metasurface made of nanogrid-patch units.^113^ This design achieved an average absorptivity of 94.2% in the 3–5 μm MIR range, with a thermal response enhanced by a factor of 2.6, significantly improving the pyroelectric current of the detector.

Low-dimensional materials, such as two-dimensional (2D) and quasi-one-dimensional (1D) structures with narrow or zero bandgaps, have recently emerged as promising candidates for MIR detection.^112^ These materials are characterized by their layered structures, composed of atomically thin nanosheets held together by weak van der Waals (vdW) interlayer forces. Among 2D materials, graphene,^115^ transition-metal dichalcogenides (TMDs) like MoS_2_ and PtSe_2_,^116^ and black phosphorus (BP)^117^ have gained attention in the MIR photodetection field due to their narrow bandgaps and excellent electronic properties.

Graphene, as a gapless 2D material, offers the potential to enable photodetectors that cover the entire IR range. However, its relatively weak absorption and high dark current limit its performance in MIR detection. Other materials, including black phosphorus (BP), black arsenic phosphorus (b-AsP), tellurene (Te), and BaTiS_3_ (BTS), have demonstrated high responsivity and detectivity in MIR detectors.^118−120^ Their layered vdW lattices result in self-terminated surfaces, making them highly compatible with silicon platforms and standard nanofabrication processes and ensuring excellent CMOS compatibility. For further insights, recent comprehensive reviews on MIR device advancements using 2D and low-dimensional structures are available in the literature for interested readers.

## CONTEMPORARY APPLICATIONS IN RELEVANT FIELDS

The following sections present a representative selection of integrated and miniaturized IR sensing systems, highlighting applications published in the past five years. These examples are discussed alongside the current requirements and future trends in each field.

### Biomedical and Clinical

Bodily fluids (e.g., blood serum/plasma, urine, saliva, cerebrospinal fluid, and tears), along with exhaled breath, contain valuable information about the presence of inorganic and organic molecules, proteins, and biochemical markers, providing critical insights into the health status. These fluids serve as alternatives to cells and tissues for disease diagnosis and prognosis, offering benefits such as minimal invasiveness, rapid and cost-effective sample collection, reduced discomfort, and straightforward processing. The analysis of bodily fluids to uncover specific biological and chemical information is becoming increasingly important. Several analytical techniques have been widely used for this purpose, including microscopy, liquid and gas chromatography coupled with several detectors, fluorescence, mass spectrometry, nuclear magnetic resonance, and vibrational spectroscopy techniques, such as Raman and MIR absorption. Each method has distinct advantages and limitations, and combining them can enhance the diagnostic reliability. Nondestructive optical waveguide IR-based sensors present several advantages over traditional techniques, including bulk liquid/gas absorption spectroscopy. These include compact and miniaturized designs, high sensitivity for small sample volumes, precise signal-to-background optimization, ready on-chip integration with microfluidics, and user-friendly operation.

The proliferation of *Escherichia coli* was investigated by Teuber et al., using a laser-based IR spectroscopy system consisting of diamond or GaAs thin-film waveguides, along with an external-cavity quantum cascade laser (QCL) emitting in the range of 5.7–6.6 μm.^121^ Bacteria were inoculated onto each waveguide and incubated for 6–36 h at 37 °C. This laser-based thin-film waveguide spectroscopy approach revealed significantly more pronounced changes in the infrared spectra compared to conventional IR-ATR spectroscopy. While long-term exposure to GaAs may have toxicological effects on microbes, short-term exposure did not appear to be harmful. This confirms the suitability of GaAs, alongside diamond, as a low-cost TFWG platform for studying live biological specimens. In another study, the quantification of bovine serum albumin (BSA) in phosphate-buffered saline (PBS) ranging from 0.1 to 100 mg/mL was performed using an ATR-based sensing system, consisting of a tunable QCL emitting between 5.3 and 12.9 μm, coupled with a thermoelectrically cooled MCT detector.^88^ Samples containing BSA were introduced onto a Ge-on-Si waveguide via flow-strip filter paper, enabling the evanescent field to interact and measuring three amide peaks. The combination of thin-film waveguides and QCLs operating in the MIR fingerprint region offers new possibilities for sensing important biomolecules for point-of-care diagnostics, leveraging the high selectivity of the MIR signatures. In a similar study, BSA and its aggregates were analyzed using the same optical platform.

**Figure 5:**
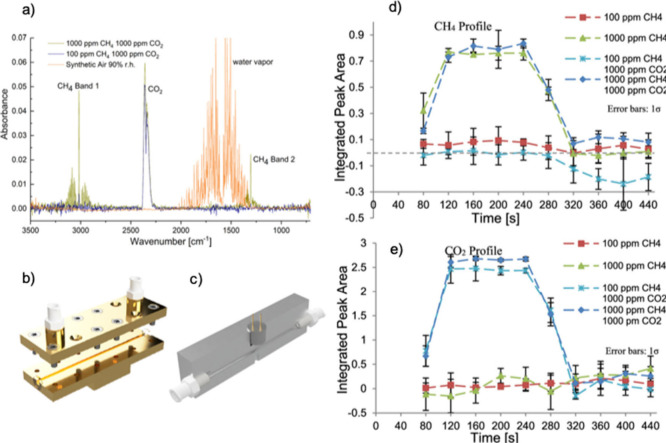
Cross-sectional view of (a) iHWG and (b) the MOX gas cell. IR absorption spectra of 1000 and 100 ppm of CH_4_ with 1000 ppm of CO_2_ in comparison to humidified air/water vapor. (a) Comparison of CH_4_ profile with and without 1000 ppm of CO_2_. (b) Time-resolved carbon dioxide profile of samples with and without 1000 ppm of CO_2_ in methane and synthetic air. [Reproduced from ref (122). Copyright 2020, American Chemical Society, Washington, DC.]

Breath analysis using MIR miniaturized sensors enables real-time analysis, cost-effectiveness, portability, and provides inherent molecular selectivity. A sensing device with combined orthogonal sensors (i.e., e-Nose and MIR) was developed using a low-volume gas cell for a commercially available semiconducting MOX gas sensor and coupled directly to a MIR gas sensor based on substrate-integrated hollow waveguide technology.^122^ This hybrid platform resulted in fast, time-resolved, and synergic detection of methane and carbon dioxide–two important biomarkers in exhaled breath–in gas samples. The FTIR spectra of both analytes are illustrated in Figure 5a. The iHWG used in this study was fabricated from brass alloy substrate (CuZn_*x*_) and the entire MOX gas flow-through cell was machined from an aluminum block (Figures 5b and 5c). The time-resolved analytical signal profiles of CH_4_ and CO_2_ are shown in Figures 5d and 5e.

**Figure 6:**
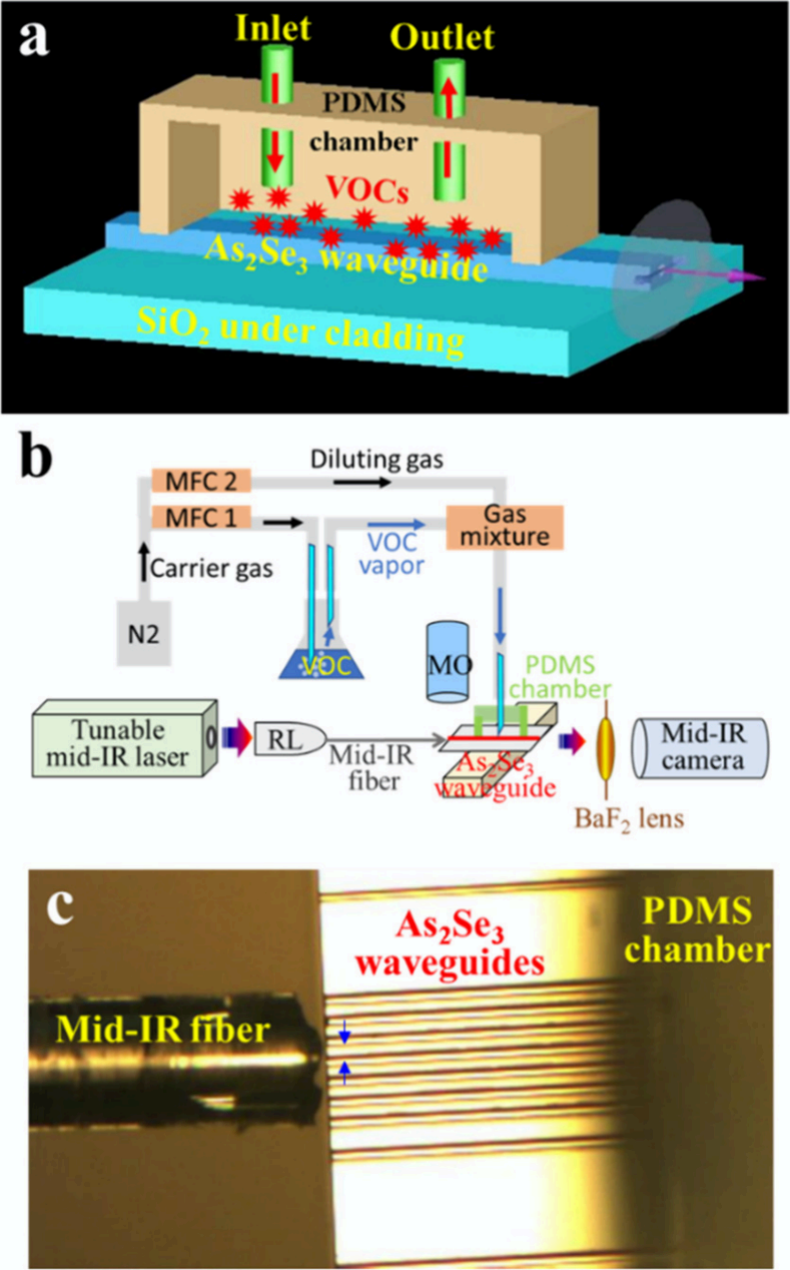
(a) Schematic of the VOC sensor that consisted of an As_2_Se_3_-on-SiO_2_ waveguide and a PDMS gas chamber. (b) Diagram of the VOC measurement system. MIR laser light was focused in a single mode fiber by a lens (RL) and delivered to the waveguide. The waveguide mode image and intensity were recorded with a camera placed after a BaF_2_ lens. VOC samples were prepared by two mass flow controllers (MFCs) and a VOC evaporation flask. MFC1 controlled the VOC flow rate, and MFC2 regulated the diluting gas N_2_. (c) A microscope (MO) was utilized to reposition the fiber and the As_2_Se_3_ waveguide. The right side of the sensor device was covered with a PDMS chamber. [Reproduced from ref (55). Copyright 2016, American Chemical Society, Washington, DC.]

The quantification of volatile compounds, such as volatile organic compounds (VOCs), is crucial in various contexts, from indoor pollution monitoring to breath analysis. Many VOCs, including ethanol, acetone, and isoprene, are biomarkers found in human exhaled breath, and their detection provides an efficient method for monitoring human health. A MIR sensor chip was developed for detecting acetone and ethanol vapors (concentration range of 0 to 100%), consisting of As_2_Se_3_ optical waveguides fabricated using microelectronic processes.^55^ The VOC sensing performance was characterized by detecting acetone and ethanol vapors at their characteristic C–H absorption bands from λ = 3.40 to 3.50 μm, with continuous detection and a response time of less than 5 s. Figure 6 presents the scheme of the proposed setup. The spectral region from 3.0 to 3.6 μm was also employed to detect acetone, ethanol, and isoprene vapors using SiN waveguides inside a PDMS chamber, coupled to a tunable QCL and photodetector, for the first time enabling on-chip MIR measurements using SiN-based materials.^64^

A miniaturized gas sensor combining MIR interband cascade light emitting diodes with the so-called substrate-integrated hollo waveguides was developed for the quantification of four relevant biomarkers that can be found in the exhaled breath (i.e., methane, isobutane, acetone and acetaldehyde).^105^ The linear regression resulted in a detectability and quantification of relevant concentrations of the biomarkers (625, 78, 40, and 59 ppmv for methane, isobutane, acetone, and acetaldehyde, respectively).

Absorption on-chip spectroscopic devices, which integrate advanced thin-film waveguide technology, powerful QCL sources, detectors, and chemometric algorithms or machine learning approaches, provide accurate biomedical and clinical analyses without the need for extensive sample processing. The biocompatibility of many MIR materials enables in-situ bioanalysis. Additionally, multiple functionalities, such as microfluidics and electronics, can be integrated into the same photonic chip, enhancing stability, miniaturization, and compactness.

### Environmental Monitoring

Monitoring a wide range of pollutants, such as organic and inorganic compounds in water and in the atmosphere, is crucial for public health and environmental protection. Typically, analysis is performed in the laboratory after a sampling step, which can lead to sample degradation or contamination. This process relies on bulky, off-line instrumentation, such as gas chromatography–mass spectrometry (GC-MS) or high-pressure liquid chromatography (HPLC) coupled with optical or MS detection. Despite their inherent accuracy and sensitivity, there remains a significant demand for technological tools that allow for rapid in-situ measurements. In this context, on-chip IR sensing devices offer the required selectivity and sensitivity, filling the gap for portable monitoring systems for environmental analysis.

Before the development of miniaturized on-chip systems, compact ATR-FTIR devices using diamond-like carbon-coated silicon wafers and chalcogenide-based waveguides were demonstrated for quantifying several organic molecules—such as benzene, toluene, ortho-, meta-, and para-xylenes, ethylbenzene, trichloroethylene, tetrachloroethylene, and naphthalene—in water samples.^123,124^ These devices enabled the quantification of analytes in the concentration range of 40 ppm to 10 ppb, with the spectral region of 6.4–14 μm providing the required selectivity. These results show that diamond- or chalcogenide-based optical microsensor devices can be tailored by coupling with appropriate laser sources to offer selective and sensitive in-situ water analysis.

A miniaturized distributed feedback (DFB) laser, emitting at a wavelength of 3.38 μm, combined with a dense-pattern multipass cell, was used for the simultaneous detection of methane and water vapor.^125^ All optical components were assembled on a 40 cm × 30 cm × 10 cm aluminum optical breadboard to achieve a small footprint. The DFB laser features reduced size and low power consumption, operating in continuous wave (CW) mode at room temperature with an output power of about 1 mW and a line width of less than 5 MHz. The sensing device achieved limits of detection of 1 and 10.4 ppm for CH_4_ and H_2_O, respectively, with an integration time of 102 s.

As an alternative to traditional silicon- and chalcogenide-based waveguide gas sensors, a novel on-chip Nb_2_O_5_ waveguide-based gas sensor was developed for methane quantification.^126^ The sensor was fabricated by using magnetron sputtering and a lift-off process. Nb_2_O_5_ offers a wide transparency range of 0.35–10 μm, making it suitable for on-chip sensing of various gases with molecular absorption fingerprints in this range, such as CH_4_ at 3.291 μm. The Nb_2_O_5_ sensor achieved a limit of detection of 348 ppm. This newly developed Nb_2_O_5_ waveguide sensor expands the family of waveguide core materials to include metal oxides, which could be advantageous for the large-scale production and commercialization of such sensors.

### Food and Agriculture

Approximately 600 million people worldwide (i.e., 1 in 10 individuals) suffer annually from foodborne illnesses caused by contaminated food.^127^ Global environmental changes, driven by urbanization and climate change, have introduced variability in crop yields and distribution, posing both direct and indirect threats to agricultural food safety. These changes disrupt rainfall patterns, alter microbial ecosystems, exacerbate the greenhouse effect, and contribute to the emergence of new plant diseases, all of which significantly impact the food production chain. At every stage (e.g., production, processing, distribution, transportation, and preparation) the contamination risks are amplified, contributing to over 200 distinct foodborne diseases. Many of these illnesses also stem from the natural production of toxic chemicals on food surfaces. Food contaminants can generally be categorized into six groups: (1) chemical contamination, such as pesticides, fertilizers, toxins, or chemical residue from cleaning products; (2) bacterial/microbial contamination, (e.g., Salmonella, Listeria, and *E. coli*); (3) viral contamination; (4) protein contamination; (5) parasite contamination; and (6) fungal contamination, such as mold or yeasts.

**Figure 7:**
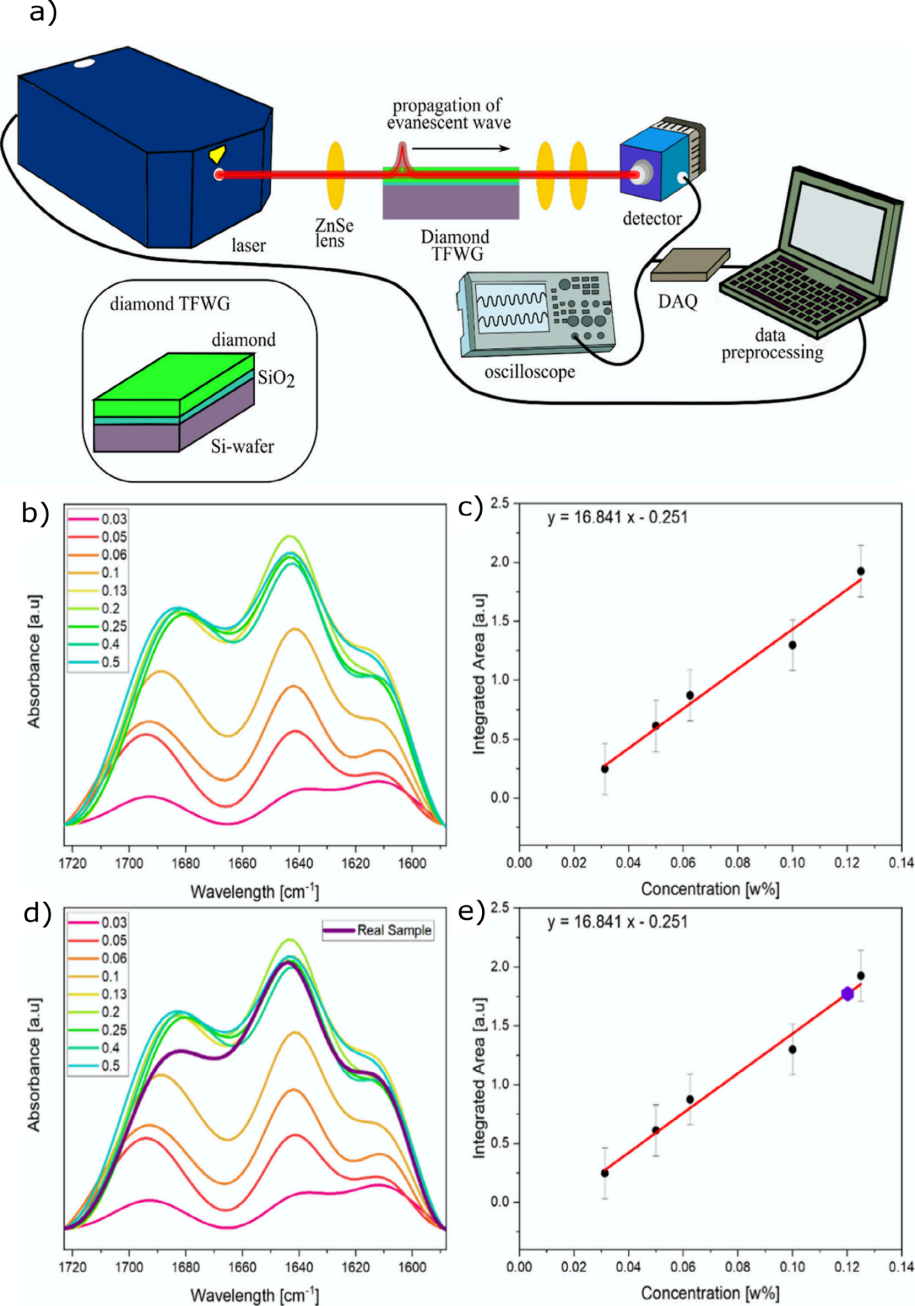
(a) Schematic of the QCL NCD TFWG sensor. A tunable QCL was used as the IR light source and a TE-cooled mercury–cadmium–telluride (MCT) as detector; (b) IR spectra of the caffeine calibration solutions; (c) calibration function; (d) calibration spectra and spectrum of the espresso sample (purple); (e) calibration function with the data point of the real-world espresso sample (purple dot). Reproduced from ref (18). Copyright 2016, American Chemical Society, Washington, DC.]

Therefore, determining the quality of foodstuffs is one of the most important priorities in food industry technology, being a mandatory step in the food production chain. Again, the MIR spectral range appears as an excellent choice due to its inherent nondestructive, eco-friendly, selective and sensitive features that enable qualitative and quantitative analysis. With the aid of the integration of broadly tunable lasers—covering spectral windows of several hundreds of wavelengths—with multireflective thin-film waveguides and miniaturized IR detectors, the development of tailored on-chip devices for food quality and agriculture applications (e.g., monitoring of greenhouse gas emissions) have been demonstrated. Recently, the application of a thin-film waveguide with a 20-μm-thick nanocrystalline diamond waveguide coupled to a tunable QCL emitting at 1754 and 1493 cm^–1^ for the qualitative and quantitative analysis of caffeine was demonstrated.^18^ Distinct and pronounced molecular signatures were obtained, facilitating the identification of caffeine even in a water background. Figures 7b–e present the IR spectra and calibration functions of caffeine using a miniaturized QCL-NCD-TFWG sensor (Figure 7a). A linear relation of 0.03 to 0.125% (w/w) was obtained with a limit of detection (LOD) of 0.019% (w/w); the method was applied to an espresso sample and the caffeine amount was determined at 0.12 ± 0.01% (w/w).

An interesting study was described by Haas et al., in which a GaAs-based waveguide as a platform for direct MIR vibrational spectroscopy was used for the detection of the mycotoxin aflatoxin B1 (AFB1).^16^ The calibration function of the analytical signal versus AFB1 was linear from 5 to 30 ppm using a GaAs-based waveguide. Very recently, the analytical performance of a MIR sensing system is presented combining a tunable interband cascade laser emitting at a wavelength range of 5.88–6.09 μm with a 6 μm GaAs/AlGaAs thin-film waveguide as the active transducer for customized evanescent field absorption analysis of food contaminants, exemplary represented by analysis of the Fusarium mycotoxin deoxynivalenol, a persistent food contaminant resulting from fungal infection. The concentration range of 0.025 to 2500 mg/mL with an LOD of 0.222 mg/mL were achieved.^128^

A variety of inorganic and organic compounds can be detected using miniaturized and on-chip IR sensors. For instance, *N*-methylaniline is an aromatic amine that can adversely affect food quality, aquatic environment, soil, and air. Rib waveguides modified by gold (Au) nanorods coated with a layer of palladium (Pd) coupled with a laser source were designed and applied for the identification of methylaniline.^129^ Potassium bicarbonate, glycerin, and hydroxide peroxide were detected using on-chip IR-based sensing via the integration of silicon-on-insulator waveguide and a tunable laser source. The proposed setup was able to detect the analytes in the 0–20% (w/w) concentration range.

The monitoring of greenhouse gases and atmospheric pollutants emitted in agriculture processes can be achieved using on-chip gas sensing by optical absorption in the MIR range, as theoretically demonstrated by Koompai et al.^130^ A Si_3_N_4_ on SiO_2_ waveguide for multigas wideband on-chip spectroscopic sensing of water vapor, carbon dioxide, nitrous oxide, ammonia, ethylene, and methane gas molecules, with a wideband operation between 2.7 and 3.4 μm was evaluated with suitable performance required for on-chip IR sensing, reaching parts per million levels for the minimum detectable concentration.

Finally, a corresponding overview of recent applications of miniaturized and on-chip devices for different analytes is presented in Table 3.

**Table 3:** Summary of Recent Applications of Miniaturized and on-Chip IR Sensing Systems

waveguide type and material	light source	application	limit od detection, LOD	ref
TFWG – SiN-on-SOI	tunable MIR laser	acetone	1.2%	(64)
TFWG – SiN-on-SOI	tunable MIR laser	ethanol	1.3%	(64)
TFWG – SiN-on-SOI	tunable MIR laser	isoprene	2.5%	(64)
				
TFWG - Ge-on-SI	QCL (5.3–12.9 μm)	bovine serum albumine (BSA)	0.1 mg/L	(76)
				
TFWG – As_2_S_3_ with PDMS chamber	nanosecond pulsed laser (3.4–3.6 μm)	acetone and ethanol vapors	25% for ethanol	(55)
				
TFWG – diamond and GaAs	external-cavity (EC) QCL (5.7–6.6 μm)	*E. coli*	–	(121)
				
TFWG – GaAs and diamond	EC-QCL	Caffeine	0.019%w/w	(18)
				
On-Chip p-InAsSbP/n-InAs Heterostructure	LED (3.46 μm)	Water and ethanol	0 – 96%	(11)
				
TFWG - Ge_28_Sb_12_Se_60_ waveguides	Laser (3–4 μm)	Cinnamaldehyde	1.09 μmol/L	(54)
				
Si Slot waveguide	DFB-ICL (3.27 μm)	methane	0.3 ppm	(131)
				
SOI Al-enhanced metamaterial waveguide	QCL (3.70–3.82 μm)	acetone, isopropyl alcohol, glycerin	972 ppm	(132)
				
Si-on-SOI	QCL (3.725–3.888 μm)	isopropyl alcohol	2%	(65)
				
Silicon-based integrating cylinder	LED (4.3 μm)	CO_2_	22 ppm	(20)
				
TFWG - Si_3_N_4_	theoretically evaluated between 2.7 and 3.4 μm	H_2_O, CO_2_, N_2_O, NH_3_, C_2_H_4_ and CH_4_	few ppm	(130)
TFWG - SiO_2_/Si_3_N_4_	QCL (4.08–4.34 μm)	CO_2_	0.1%	(63)
				
TFWG – Nb_2_O_5_	ICL (3.29 μm)	methane, propane, ethane and butane	346 ppm	(126)
				
Al-iHWG	ICL-LED	methane	625 ppm	(105)
Al-iHWG	ICL-LED	isobutane	78 ppm	(105)
Al-iHWG	ICL-LED	acetone	40 ppm	(105)
Al-iHWG	ICL-LED	acetaldehyde	59 ppm	(105)

## CHALLENGES AND PERSPECTIVES

On-chip IR spectroscopy focuses on integrating multiple functionalities, such as microfluidics and microelectronics, into a single photonic integrated circuit while delivering enhanced stability, high analytical sensitivity, and ease of operation. However, achieving seamless integration of optical sensors with other technologies is challenging due to the diverse fields involved, including solid-state physics, electronics, photonics, materials science, and engineering. The combination of high-power tunable light sources, thin-film waveguides, and highly sensitive, miniaturized detectors has significantly improved signal-to-noise ratios compared to those of conventional FTIR systems that rely on bulky internal reflection elements (IREs).

Advances in the design and fabrication of thin-film waveguides are critical to driving the next generation of infrared (IR) sensing technologies. The exploration of a variety of optical materials has enabled the use of the broad mid-infrared (MIR) spectral range (2.5–25 μm), expanding the operational scope of IR devices. Nonetheless, fabricating homogeneous, robust, and mechanically stable thin-film waveguides remains a significant challenge. Current deposition techniques are often time-consuming, require the use of strong chemicals, lack reproducibility, and involve multiple steps. Balancing the tradeoff between simpler fabrication methods and the choice of waveguide materials is therefore essential. Innovations in the design of photonic bandgap fibers and substrate-integrated hollow waveguides have reduced the footprint of gas-based sensors, facilitating easy integration with light sources and detectors. However, further developments are required to enhance the sensitivity for applications in environmental monitoring, clinical diagnostics, and food analysis.

The implementation of tunable laser sources with broad IR spectral coverage and high light output has substantially improved the analytical sensitivity and broadened the applicability. Nevertheless, the high cost of assembling IR-based analytical devices remains a significant barrier compared to those of ultraviolet (UV)-, visible-, and near-infrared (NIR)-based techniques. A cost-effective alternative is the use of MIR light-emitting diodes (LEDs) as light sources. However, their optical power output, typically in the μW to mW range, is considerably lower than that of LEDs operating in the visible and NIR ranges. Furthermore, the broad emission spectra of LED-based sensors increase their susceptibility to absorption overlap between analytes and interferences, resulting in reduced selectivity for real-world samples.

Despite these challenges, the MIR spectral range offers unparalleled advantages in terms of selectivity, sensitivity, and its nondestructive, label-free nature, highlighting its potential for ATR- and absorbance-based methods. Recent advancements in MIR technology have driven the development of miniaturized on-chip sensing solutions for diverse applications. This Perspective discusses key progress in areas such as light sources, waveguides, and detectors. With recent advancements, a wide range of applications in diverse analytical chemistry fields are anticipated in the coming years. The future of advanced on-chip IR sensors is just around the corner.
